# Tidying up white matter: Neuroplastic transformations in sensorimotor tracts following slackline skill acquisition

**DOI:** 10.1002/hbm.26791

**Published:** 2024-11-11

**Authors:** Karl Koschutnig, Bernhard Weber, Andreas Fink

**Affiliations:** ^1^ Department of Psychology, MRI‐Lab Graz University of Graz Graz Austria; ^2^ Department of Psychology University of Graz Graz Austria; ^3^ Present address: MRI‐Lab Graz, University of Graz, Kopernikusgasse 24 Graz Austria

**Keywords:** brain plasticity, fixel‐based analysis, motor learning, sensorimotor functions, white matter morphology

## Abstract

This study investigated changes in white matter (WM) morphology following complex motor learning, that is, the learning to walk a slackline. A sample of young adults from the general population underwent brain imaging before the slackline intervention, after successful learning, and after a subsequent follow‐up period by applying state‐of‐the‐art measures for the assessment of micro‐ and macrostructural characteristics of WM fiber tracts (voxel‐based and fixel‐based). A randomly assigned control group (CG) was scanned at the same time points of assessment but received no intervention over the study period. Learning to walk a slackline resulted in manifold changes in WM morphology: (1) Whole brain fixel‐based analyses revealed robust increases in the fiber cross‐section in bundles closely associated with sensorimotor functions (e.g., superior longitudinal fasciculi, corticospinal tract); (2) The neurite orientation dispersion and density imaging (NODDI) parameters showed widespread decreases in overlapping fiber bundles. In the CG, no time‐related WM changes were apparent at all. This well‐controlled longitudinal intervention study provides substantial new evidence that learning a complex motor skill modulates fiber organization and fiber density in sensorimotor tracts.

## Graphical abstract

This randomized longitudinal study investigates training‐induced changes in white matter morphology after a highly challenging motor learning task (slackline). Changes were found solely in the intervention group, demonstrated in NODDI (green box) and Fixel‐related metrics (yellow box).

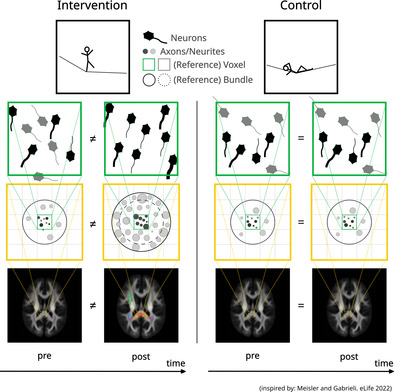

## 1 INTRODUCTION

After birth, humans need about seven years of practice to achieve complete balance control. While childhood covers the most significant improvements, exposure to specific postural training can also improve adult balance control. Postural control and balance are “a whole‐brain phenomenon” (Surgent et al., 2019, p. 249) and are usually not restricted to some isolated regions of the brain. However, some brain regions, including the hippocampus (Hüfner et al., 2011), the basal ganglia (Taubert et al., 2010), and the inferior partial cortex, seem crucial in most studies.

There is compelling evidence from experiments with different experimental approaches that “brain plasticity” is mainly based on synaptic plasticity (Abbott & Nelson, 2000). Yet, converging evidence suggests () that myelin plasticity is also a hot candidate to explain experience‐related reshaping of structural and functional characteristics of the brain (Lakhani et al., 2016; Sampaio‐Baptista et al., 2013; Scholz et al., 2009).

Several studies have demonstrated that motor learning interventions evoke white matter (WM) neuroplasticity. These alterations have been observed through various interventions such as aerobic exercise and learning unconventional skills like unicycling (Frizzell et al., 2020; Weber et al., 2019). Learning a new motor skill can prompt structural modifications in WM pathways relevant to the task, which may indicate increases in myelination associated with learning (Sampaio‐Baptista et al., 2013). Furthermore, a recent study offers insights based on animal and computational models (Bacmeister et al., 2022). The authors demonstrated that myelin plasticity induced by behavior is concentrated in axons activated during learning, potentially leading to the retraction of myelin sheaths during the learning process and the subsequent addition of newly formed myelin.

An expanding body of literature also suggests that brain plasticity in WM can occur over remarkably brief periods. For instance, studies by Huber et al. (2018), Sagi et al. (2012), and Taubert et al. (2010) have highlighted this phenomenon. Notably, Hofstetter et al. (2013) observed changes in diffusivity indices induced by a learning task within a mere two‐hour timeframe in humans. Therefore, diffusion‐weighted imaging proves invaluable for scrutinizing both short‐ and long‐term alterations in WM.

Most previous work on WM plasticity is based on diffusion MRI (dMRI). This approach uses a diffusion tensor model (Basser & Pierpaoli, 1996) to estimate specific WM metrics, including fractional anisotropy (FA), radial diffusivity (RD), and mean diffusivity (MD). This model is a powerful and sensitive tool for investigating microstructural changes in WM. Still, several limitations prevent a complete understanding of the basics of WM plasticity. This model can only estimate one fiber population within each voxel. In the presence of kissing and crossing fibers, the WM tissue microstructure is probably falsely estimated, and measurements potentially lack tissue specificity (Jelescu et al., 2016).

More sophisticated models may provide an exciting new perspective to learn more about the functional mechanisms implicated in experience‐induced brain changes. One branch of models to characterize the complexity of cerebral tissue is subsumed under the term compartment model, where the diffusion‐weighted signal is decomposed into separate compartments. One of these multicompartment models provides information about the neurite density index (NDI) and orientation dispersion index (ODI), which are sensitive to axonal packing and tract coherence (Zhang et al., 2012). For example, age‐related variance could be better expressed with NODDI than with the tensor model (Venkatesh et al., 2020). Kamiya et al. (2020) summarize the potential biological meaning of different NODDI parameters in clinical research and provide a quick overview. For example, an NDI decrease in WM is thought to reflect axonal degeneration, while the NDI increases should indicate axonal swelling or adaptive axonal regrowth. The ODI decreases are also believed to reflect axonal regrowth, whereas the ODI increases may reflect axonal disorganization or increased extra‐axonal space. These biological mechanisms have been identified in clinical research, and it is presently unclear how they relate to samples from the general population. They nevertheless provide an important framework of ideas potentially underlying the observed changes in NODDI parameters.

Another way to overcome the tensor model's limitations is to use higher‐order diffusion‐weighted imaging models to estimate the fiber orientation distribution (FOD) regardless of the number of fiber bundles projecting through one voxel (Behrens et al., 2007; Tournier et al., 2004). This approach has recently been expanded to multishell, multi‐tissue constrained spherical deconvolution (CSD) to increase the FOD's precision (Jeurissen et al., 2014). Nevertheless, to make meaningful estimates of WM plasticity, it is also important to establish quantitative metrics that can independently characterize changes in specific fiber populations. This can be achieved with a recent technique (Raffelt et al., 2015, 2017) called fixel‐based analysis (FBA). A “fixel” is defined as a specific fiber population within a voxel. Within this framework, it is possible to estimate changes in (a) the fiber density (FD), (b) the cross‐section (FC), and (c) a combined measurement of both metrics (FDC). FC indicates the cross‐sectional area of neural fibers, providing information about the size and integrity of these fibers. The FD is essentially not sensitive to myelin (Raffelt et al., 2011) and approximately (linearly) proportional to intra‐axonal volume (Dhollander et al., 2021). FDC combines information on the density and cross‐sectional area of fibers, offering a comprehensive view of the structural characteristics of neural pathways. Fixel‐based analysis has been successfully applied in different clinical studies (Dimond et al., 2019; Li et al., 2020; Verhelst et al., 2019). Non‐clinical studies in this context investigated aging (Choy et al., 2020) and development in late childhood (Genc et al., 2018). Still, no study investigated the influence of comprehensive postural training with fixel‐based analysis in a longitudinal study design.

This randomized longitudinal study investigates training‐induced changes in WM morphology after reaching a predefined ability level in slacklining and after a subsequent follow‐up period by applying different white‐matter‐related analysis strategies (voxel‐based and fixel‐based). A cohort of 60 participants was assigned to an intervention group and a control group (CG). Each participant underwent an MRI at three different assessment times. The primary research question was whether a highly challenging postural intervention (slacklining) leads to short‐term changes in white‐matter morphology. More specifically, slackline‐induced changes were characterized by (a) fixel‐based metrics and (b) metrics from neurite orientation dispersion and density imaging (NODDI). Because both models enable a more comprehensive analysis of WM changes, we hypothesize that this approach can be more sensitive to training‐induced changes than the commonly used tensor model.

## 2 RESULTS

### 2.1 Slackline training

Slacklining is a well‐established and easily applicable task that requires participants to learn novel movements involving visual–spatial coordination. This complex and challenging task may constitute a promising model for studying experience‐dependent changes in WM morphology. A professional trainer guided the slackline training with one training unit (about 90 min) weekly for up to 3 weeks. The maximum training duration for each subject was 270 min. The training took place indoors using two 12‐m‐long slacklines (width: 3.7 cm, elongation at 10 kN: 5%), each suspended at a height of 40 cm and supported in the middle by a gymnastics box. This setup facilitated training for up to four participants simultaneously. The slackline training ended after a participant had all the following criteria met: (a) easily get on the slackline, (b) walk 6 m on the slackline without a break, and (c) maintain balance on the slackline for a minimum of 2 min.

**FIGURE 1:**
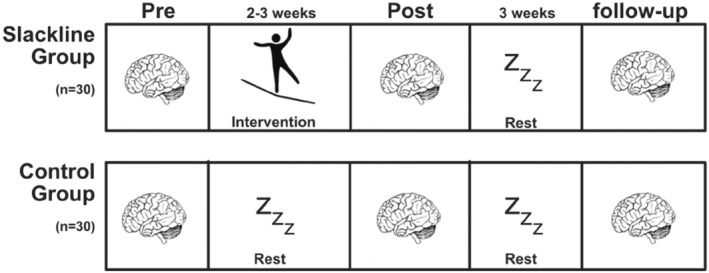
Study design. Both groups underwent a baseline assessment session (pre‐test) involving brain imaging (MRI). The slackline intervention took about 2–3 weeks, and the time between the pre‐test and the post‐test for the CG was matched to that of the SG. MRI was assessed again after reaching the predefined criteria of a certain slackline ability (post‐test). After a further 3 weeks of no training in both groups, a follow‐up assessment was performed.

The study design involved three magnetic resonance (MR) measurements. Firstly, an initial assessment was conducted before the commencement of the training program to establish baseline data. Following this, a second MR measurement was administered within 24 h once participants had reached the predefined criteria of proficiency in slacklining. Finally, a third follow‐up MR assessment was scheduled 3 weeks after the completion of the intervention to evaluate the persistence of any observed neuroplastic changes over time (see Figure 1). Sixty subjects were randomly assigned to the slackline intervention group (SG) receiving the intervention between the pre‐test and the post‐test, or the CG receiving the intervention after the study period (see Figure 6).

### 2.2 Measuring WM plasticity

To test training‐induced changes in WM morphology, we applied a comprehensive assessment and analysis approach: (1) fixel‐based analysis, (2) NODDI, and (3) a tensor‐based model. All reported tracts are based on the JHU WM Tractography Atlas. First and foremost, time‐related changes in these measures were only found in the slackline group (SG), most apparent directly after the slackline intervention. Table 1 shows an overview of all metrics.

**TABLE 1:** Overview of significant whole‐brain changes over time for fixel‐based analysis, tensor‐based analysis, and NODDI in the SG.

		Pre–post		Pre follow‐up	
		Decrease	Increase	Decrease	Increase
FBA					
	FC	—	sign.	—	sign.
	FD	—	sign.	—	—
	FDC	—	sign.	—	—
Tensor					
	FA	sign.	—	—	—
NODDI					
	NDI	sign.	—	—	—
	ODI	sign.	—	sign.	—

*Note*: No significant changes were found between post and follow‐up. No changes were found in the CG. The significance threshold was set to (*p*_FWE_ < .01).

Abbreviations: FA, fractional anisotropy; FBA, fixel‐based analysis; FC, fiber‐cross‐section; FD, fiber‐density; NDI, neurite density index; ODI, orientation dispersion index.

### 2.3 Whole‐brain fixel‐based analysis

**FIGURE 2:**
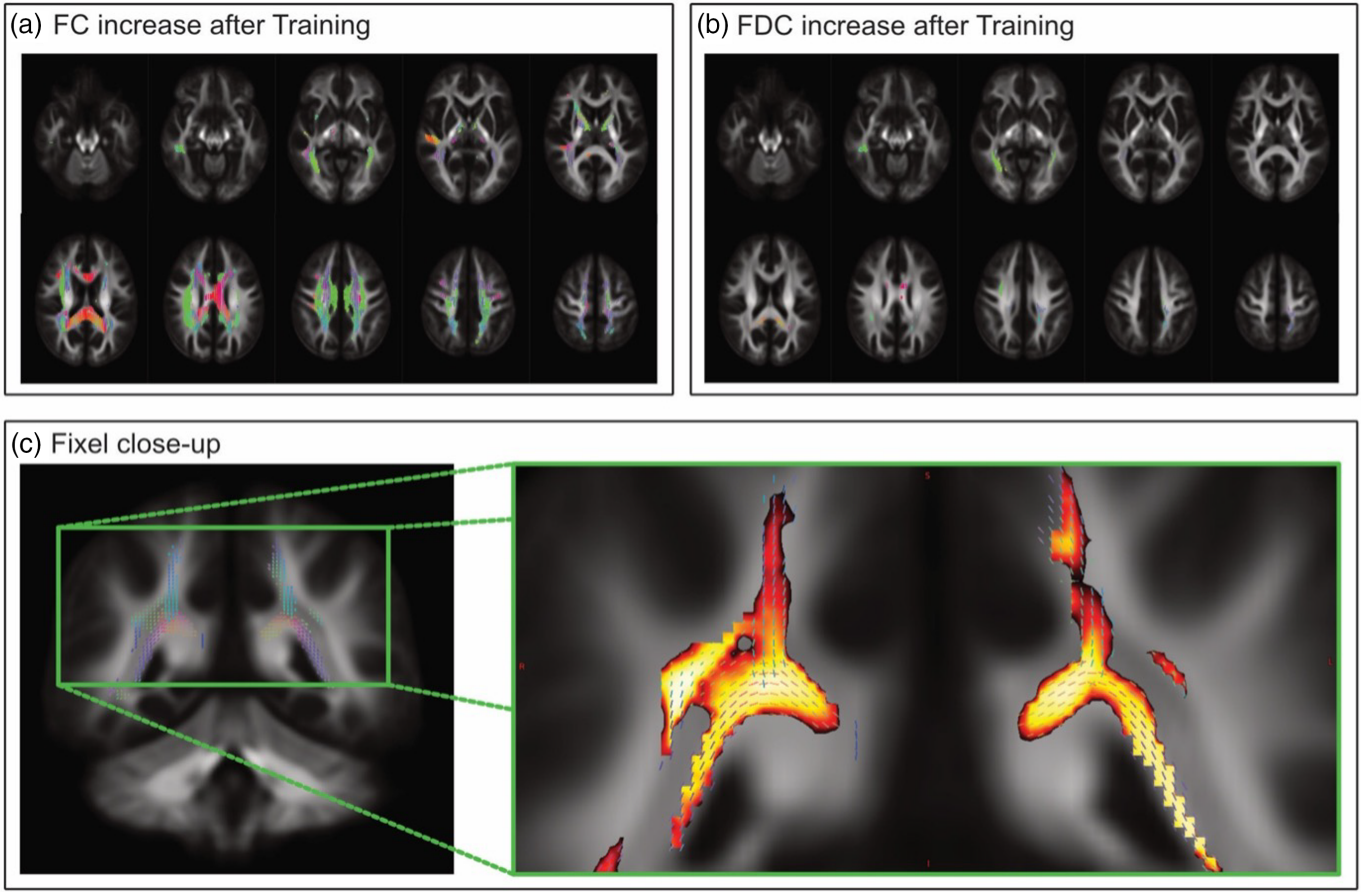
Fixel‐based results. All shown results were found after slackline intervention and only in the intervention group (*p*_FWE_ < .01). (a) Widespread whole‐brain increases in fiber‐cross‐section (FC). (b) Equally strong but more focally narrowed increases in the combined fibre‐density and cross‐section (FDC) measure. Color‐map (a–c) represents the fixel‐direction rather than the significance value. Changes in FC were more pronounced and associated with a wider network than changes in FDC. Appreciating the multidimensional analysis of the FBA, we combined the fixel direction ((c)—left‐hand side) with the actual significance level of the statistical outcome between the pre and post of the FC ((c)—right‐hand side).

We investigated longitudinal changes in white‐matter plasticity with the novel framework of fixel‐based analysis. This included all three fixel‐based metrics: fiber cross‐section (FC), FD, and the combination of both (FDC). We first computed the interaction between group and time (see Appendix S1). As expected, significant differences were only found in the SG and between the pre‐test and the post‐test. Figure 2 shows whole brain streamline segments associated with fixel‐derived indices significantly increased after the slackline intervention. Only fixels with a stringent correction for multiple comparisons are shown (*p*_FWE_ < .01). The color maps in Figure 2a–c represent the fixel‐direction rather than the significance value. Changes in FC were more pronounced and associated with a wider network than changes in FDC. Appreciating the multidimensional analysis of the FBA, we combined the fixel direction (Figure 2c, left‐hand side) with the actual significance level of the statistical outcome between the pre and post of the FC (Figure 2c, right‐hand side).

**FIGURE 3:**
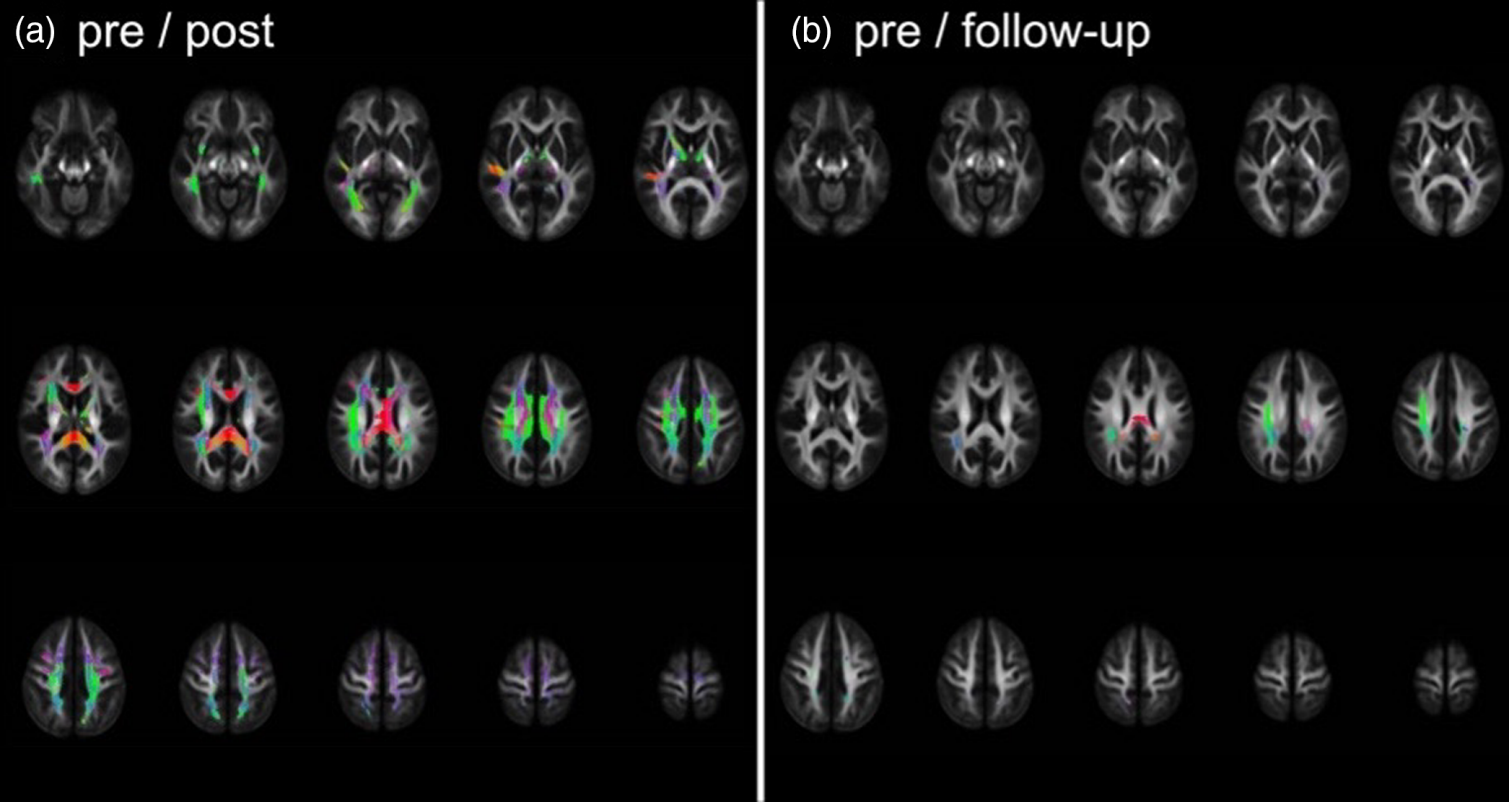
Whole‐brain increases in fiber‐cross section. (a) Widespread changes of FC right after the slackline intervention and (b) focal changes between pre and follow‐up indicating more long‐term changes. The color‐coding of the white matter fiber tracts is: Green: Anterior–posterior, red: Left–right, blue: Inferior–superior.

Although all three fiber metrics increased, the most significant changes were found after the slackline intervention in the fiber‐cross section (Figure 3). A massive cluster of almost 90.000 voxels and four smaller distinct clusters showed higher FC at the second time point in both hemispheres, including the following tracts: fasciculus arcuatus, superior and longitudinal fasciculus, anterior and superior thalamic radiation (TR), the corticospinal tract and the forceps major (FM). In addition, we found a focal increase in FD parameter in two different clusters. The first cluster comprises the left middle cerebellar peduncle and parts of the left corticospinal tract. The second cluster is about a third of the size of the first cluster and showed increases in the same regions but in the right hemisphere (see Section 3 for details). The combined measurement (FDC) showed the most pronounced increases in a cluster part of the striatum and the posterior TR in the right hemisphere. A smaller cluster revealed increases in the right superior corona radiata. There were also minor changes in the left corpus callosum and the left posterior corona radiata.

To underscore the substantial alterations observed within the FC‐metric, we depicted the increases between pre‐and post‐intervention, as well as between pre‐intervention and follow‐up, in a whole‐brain slice‐view illustrated in Figure 3. The combined view (c) portrays inclusive increases from pre to post and pre to follow‐up, providing visual evidence of widespread WM plasticity following the slackline intervention. Furthermore, to enhance the comparability of the slackline training's impact on fixel‐derived metrics, we included the effect size (Cohen's *d*) for changes between pre‐and post‐intervention in Figure 2.

### 2.4 Whole‐brain voxel analysis

#### 2.4.1 Neurite orientation dispersion and density imaging

**FIGURE 4:**
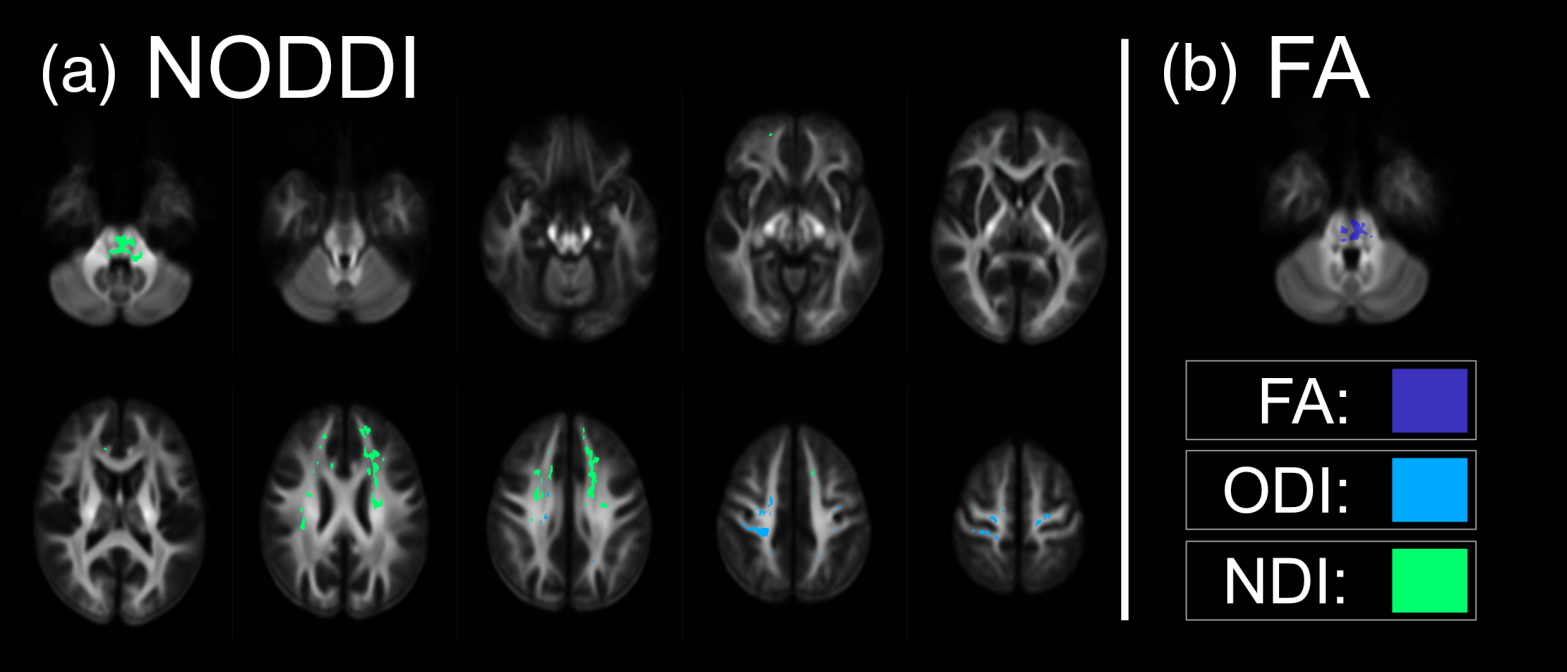
Changes in voxel‐based metrics. Both plots refer to decreases after the slackline intervention only for the intervention group. (a) Significant decreases were found in the NODDI‐parameter neurite density index (NDI) as well as in the orientation dispersion index (ODI). Slice‐view clearly demonstrates that changes in NDI are far more pronounced than for the ODI. (b) FA, in turn, showed relatively small and very focal decreases in the brain stem.

The whole‐brain analysis of the NODDI‐metrics, representing the NDI and the ODI, showed widespread decreases after the slackline intervention, as depicted in Figure 4a. Looking at the spatial extent, these changes are far more pronounced than in the tensor‐based FA metric (Figure 4b), but on the other hand, substantially smaller than the fixed‐based results. A decrease in NDI could be found in 11 distinct clusters ranging from a spatial extent of 2265–69 voxels. The biggest cluster comprises the anterior TR and the frontal aslant tract in the left hemisphere. Other involved tracts are the corticospinal tract, the superior longitudinal fasciculus, the fasciculus arcuatus, the FM, and the middle cerebellar peduncle. It is worth noting that the orientation dispersion also showed changes between pre and follow‐up, indicating short‐ and long‐term changes.

#### 2.4.2 Tensor‐derived metrics

We also analyzed longitudinal changes in tensor‐based metrics to compare the new fixel technique with more conventional approaches. To this end, we calculated FA metrics based on a tensor model. Differences in FA were only found directly after the slackline training in the intervention group. Surprisingly, our findings indicate a decrease in FA in a very focal part of the brain stem (Figure 4b), drawing a completely different picture than the fixel‐analysis. No differences could be found between pre and follow‐up in the intervention group.

#### 2.4.3 Regions‐of‐interest

**FIGURE 5:**
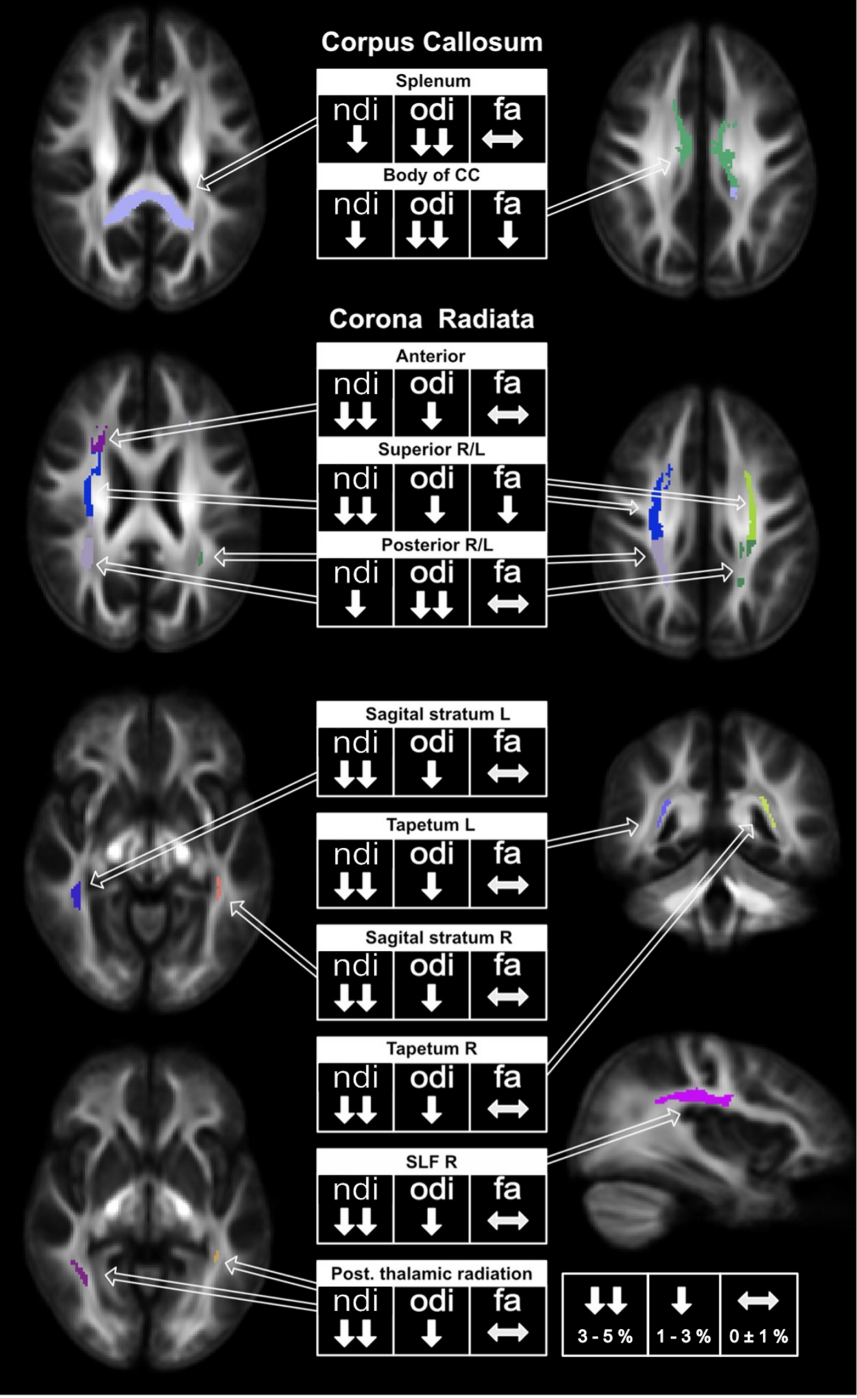
ROI‐analysis. Region‐of‐interest analysis based on fiber‐cross‐section results to further investigate differences between FBA, NODDI, and DTI. Mean values of other metrics were computed within these regions. Arrows indicate changes: Upward arrows (↑) for increases and downward arrows (↓) for decreases. SLF, superior longitudinal fasciculus.

To examine WM changes using different methodological approaches more comprehensively, we utilized a fixel‐based analysis to define regions‐of‐interest (ROIs) that showed significant differences in fiber‐cross sections between pre‐and post‐intervention (Figure 5). The mean values of two NODDI parameters, the NDI, ODI, and FA, were extracted from these regions. This approach enabled a better understanding of the differences or commonalities in the different modalities. Our findings revealed a clear decrease in NODDI parameters in the defined ROIs after the slackline intervention, with higher decreases observed in the NDI. The ODI also showed a similar pattern. In contrast, changes in FA were inconsistent, with no significant changes observed in most ROIs, and only some ROIs showed decreases.

## 3 DISCUSSION

Acquisition of slackline walking skills led to significant alterations in the morphology of WM. Whole‐brain fixel‐based techniques unveiled robust short‐term enhancements in the fiber cross‐section of various fiber tracts. These enhancements were particularly prominent in the fasciculus arcuatus, superior and longitudinal fasciculi, TR, corticospinal tract, and FM. Simultaneously, we observed widespread reductions in NODDI parameters within overlapping fiber bundles. Critically and importantly, there were no WM changes over time in the CG. Notably, WM changes were most pronounced immediately following the slackline intervention. During the follow‐up assessment, only a small fraction of these effects persisted (refer to Figure 3). This unequivocally underscores the dynamic nature of post‐training WM modifications, with a rapid return to baseline when participants discontinue their training regimen.

Research on the adaptability of neurons in the human brain has primarily focused on grey matter morphology and functional connectivity. However, it has been recognized more recently that WM structures are also highly dynamic and responsive to environmental demands. The conventional method of analyzing brain scans using diffusion tensor imaging (DTI) provides a limited spatial resolution of about 2 mm^3^. Walhovd et al. (2014) recently estimated the cellular composition of WM in an average human brain, which includes approximately 5 million axons, 700,000 oligodendrocytes, 180,000 astrocytes, 52,000 oligodendrocyte precursor cells (OPCs), and 76,000 microglia. These cell types are likely to respond differently to changes in neuronal demands, highlighting the importance of using WM analysis techniques that can reveal precise cellular changes. Fixel‐based analysis is a promising approach in this direction, addressing the limitations of the commonly used tensor model.

It is necessary to employ a diverse and complementary array of methods to capture these dynamic transformations. Our study achieved this by utilizing fixel‐based analysis and Neurite Orientation Dispersion and Density Imaging. In doing so, we specifically scrutinized fiber pathways exhibiting notable alterations in fiber cross‐section and examined NODDI parameters within these precise regions. As illustrated in Figure 5, it becomes readily apparent and remarkably consistent that both NODDI parameters (the NDI and ODI) display a reduction following the slackline intervention. The proposed NODDI model (Zhang et al., 2012) quantifies the complexity of neural tissue. In the context of pathological conditions, a decline in complexity has been linked to the onset of specific diseases, such as multiple sclerosis (Grussu et al., 2017). A decrease in complexity may signify morphological changes in individual axons or a deficit in collateral branching. However, our study diverges from this perspective regarding WM plasticity associated with motor skill acquisition, which differs from conditions implicated in neurodegenerative disorders. Within the domain of skill acquisition, a reduction in WM complexity could be viewed as a targeted refinement or restoration process. These processes might be necessary to facilitate more effective long‐term learning during the initial phases of skill acquisition. Importantly, our research demonstrates that these processes lose significance during the follow‐up measurement. This distinction sets our findings apart from studies on neurodegenerative conditions, where a stable decrease in complexity is typically observed over time. Remarkably, it can also be posited that these alterations coincide with a restoration process.

Widespread increases in fixel‐based parameters paralleled changes in NODDI parameters. Fixel‐based analysis (FBA) is a relatively new method that allows each unique‐oriented fiber population to be assigned a set of distinct metrics (FD, FC, and FDC). Therefore, metrics in one voxel are not limited to only one value (as in NODDI); rather, multiple fiber populations can be estimated. The utilization of FBA has been steadily increasing in recent years, reflecting a growing trend in research methodologies within this field. For example, Kristensen et al. (2023) showed that fiber density and fiber‐bundle cross‐section of the corticospinal tract are distinctly linked to psychosis‐specific symptoms in antipsychotic‐naïve patients with first‐episode schizophrenia. More closely related to this manuscript, fiber‐specific structural properties relate to reading skills in children and adolescents (Meisler & Gabrieli, 2022). In addition, very similar to this study, reading skills were inversely associated with the NODDI parameters in that study, which could likewise signal more efficient information processing.

Previous research in the field of FBA has predominantly centered on investigating pathological conditions (Li et al., 2020; Verhelst et al., 2019) or age‐related degenerative processes (Han et al., 2023). Most of these studies have reported decreased FBA metrics, often associated with neural atrophy. This reduction in fiber density, a parameter closely tied to intra‐axonal volume, has been linked to either a decrease in fiber diameter or a general decline in the number of fibers. In one of the rare studies employing FBA in nonpathological contexts, (Bianco et al., 2023) found an increase in fiber density within the fronto‐basal ganglia‐cerebellar circuitry associated with improved procedural learning performance. The authors posited that this increase in fiber density is likely connected to enhanced information processing capacity.

The intervention in our study modulated WM morphology in pathways closely related to visuospatial functions, like the superior longitudinal fasciculus and the FM, as well as in pathways associated with sensorimotor functions, such as the corticospinal tract and the arcuate fasciculus. Partially assigned to both networks/systems, the TR was also affected. The TR is reported to be crucial for movement generation and self‐action monitoring (Sommer, 2003), along with relaying visual input information from the eyes to the occipital cortex (Filley, 2002).

A fiber bundle that was especially sensitive to the slackline intervention was the FM, a fiber bundle connecting both occipital cortices and linking the parietal lobe and the visual cortex (Caminiti et al., 2009; Hofer & Frahm, 2006). Wang et al. (2020) refer to findings highlighting the essential role of the FM in regulating the efficiency of visual attention. Visuospatial processing demands appear to be of special relevance in learning to walk a slackline since the participant has to continuously monitor their position in space (Muiños & Ballesteros, 2021). This pertains to the connectivity of the dorsal visual pathway, crucial for spatial information analysis (Caminiti et al., 2009; Hofer & Frahm, 2006). In a similar vein, (Ceschin et al., 2015) highlight the FM's role in regulating visual attention efficiency.

This study revealed that complex motor learning results in robust increases in the fiber cross‐section, paralleled by lowering the complexity of neurite orientation. The most important benefits of this study could be seen on the one hand in the important fact that these novel metrics of WM morphology were studied in nonpathological conditions involving motor skill acquisition in a sample of young adults from the general population. On the other hand, this study evidently demonstrates that these measures provide important new insights into experience‐dependent changes in WM morphology, which cannot be adequately represented with conventional DTI methods. We very much hope that this study stimulates new research in this field, focusing on the cellular and biochemical mechanisms underlying the observed changes in WM morphology. One potential method for future studies of cellular changes is LIONESS (live information‐optimized nanoscopy enabling saturated segmentation) (Velicky et al., 2023), which allows 3D reconstruction at a synapse level, incorporating molecular activity and morphodynamic information.

This study also has some important, broader implications that should be briefly mentioned. (1) This study contributes to a nascent research field at the interface of Sports and Human Movement Science, Neuroscience and Psychology, showing that engagement in physical activity modulates functional and structural characteristics of the brain. In this particular context, relevant studies in this field are challenged by the exciting research question of how these brain changes drive related affective and cognitive functions. This appears to be especially relevant for activities such as slacklining or dancing, which involve a plethora of visual‐coordinative and ‐motor demands that could be beneficial also for visuospatial cognitive functions (Voelcker‐Rehage & Niemann, 2013). (2) Another important finding of this study was that the observed intervention‐related changes in WM morphology were most pronounced immediately following the slackline intervention, at follow‐up assessment only a small portion of effects remained. This finding supports similar evidence from both the cardiovascular and the visuomotor fitness domains showing that brain changes after physical activity interventions tend to return toward baseline after participants are no longer engaged in the training (Fink et al., 2021; Thomas et al., 2016; Weber et al., 2019). (3) This study also exemplifies the need to use different parameters of brain changes within one and the same experimental design instead of focusing on single measures. For example, the use of fixel‐based and NODDI parameters in this study yielded a quite comprehensive pattern of results, which could not have been achieved using single measures such as the FA. And (4) future research is also challenged by identifying possible factors contributing to WM plasticity, such as participant's sex (Kirby et al., 2024). Focusing on potential gender effects in this context would also be especially exciting in light of recent behavioral evidence on sex differences associated with the benefits of physical activity (Ji et al., 2024).

## 4 CONCLUSION

Novel fixel‐based metrics and NODDI parameters derived from diffusion‐weighted brain imaging have mostly been investigated in neurodegenerative disorders studies. In this study, we applied these novel measures of WM morphology to assess brain plasticity following complex motor learning in a sample of young adults from the general population. After participants had learned to walk the slackline, they showed substantial increases in the fiber cross‐section of bundles closely linked to sensorimotor functions, paralleled by robust decreases in the neurite density and orientation dispersion indices. These findings appear to indicate a state in which initially more uncontrolled proliferated neural tissue has undergone complex refinements towards more efficiency and automaticity, facilitating effective balance and postural control.

## 5 MATERIALS AND METHODS

### 5.1 MRI data acquisition

**FIGURE 6:**
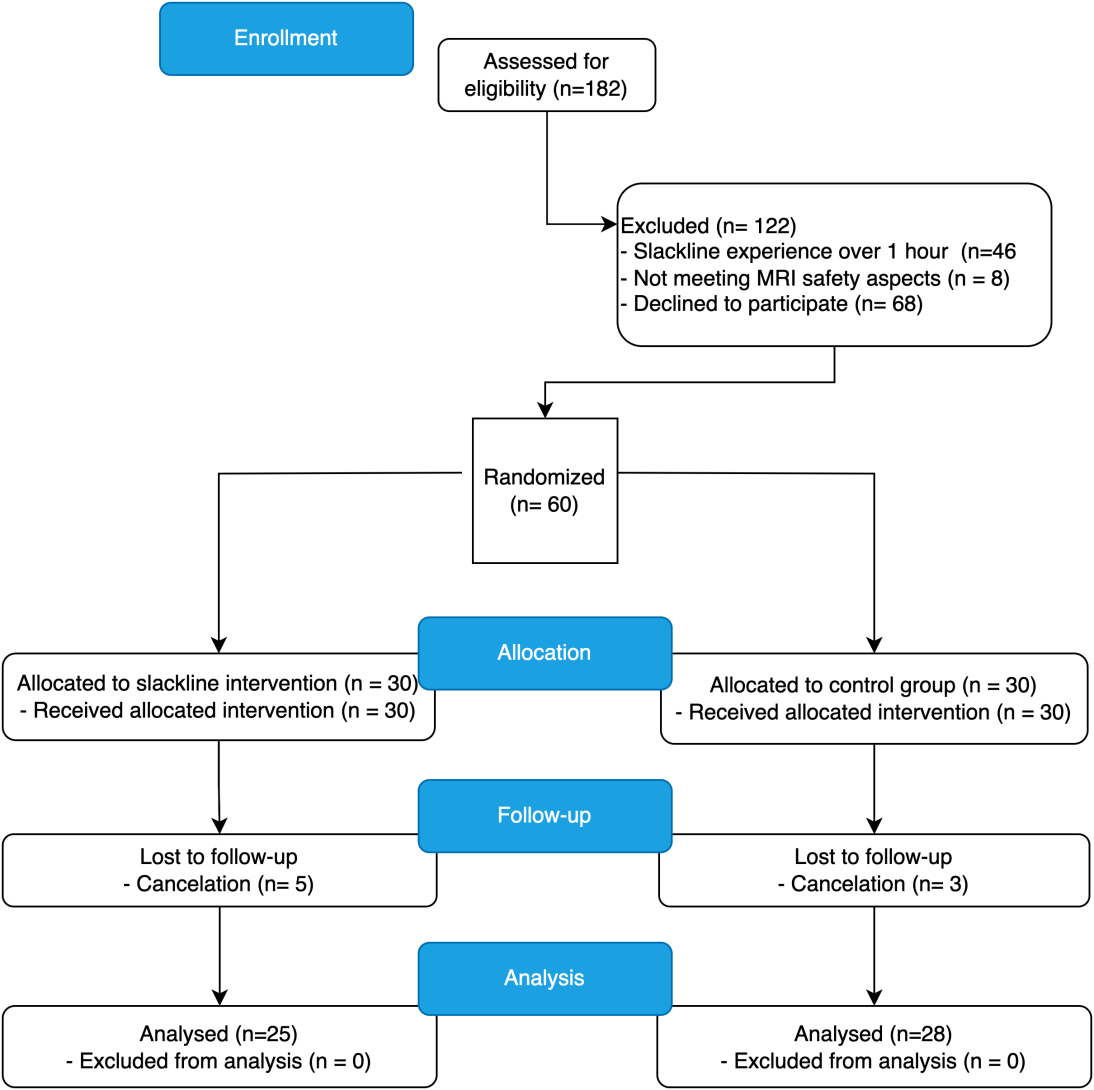
Flow chart of participants.

Totally 182 participants indicated their interest in participating in this study (see Figure 6). These participants were screened via an online survey to eliminate participants with a cumulative slacklining pre‐experience of over 1 h. After ensuring MRI safety, 136 participants were eligible to participate in the slackline intervention study. Of these, 60 participants were randomly assigned to either the slackline intervention group (SG) or the CG. The SG received the intervention between the pre‐test and the post‐test. The CG had the opportunity to receive the same training after completing the study. During the study, seven participants cancelled their attendance, resulting in a final sample size of 25 participants in the SG (15 females, age: mean = 22.8, SD = 2.7) and 28 in the CG (16 females, age: mean = 25.4, SD = 3.01). The CG participants were significantly older, with a mean difference of approximately 2.5 years (*t*(51) = 3.27, *p* = .002). There were no significant differences in sex (chi‐square (1, *N* = 53) = 0.044, *p* = .833) or slackline‐pre‐experience (Mann–Whitney *U*: *U* = 315, *p* = .512) between the two groups. During the study, participants were instructed to maintain their usual physical activity routine and refrain from balance training.

MRI data were acquired at the MRI‐Lab Graz, Austria, on a 3.0T Siemens Skyra (Siemens, Healthineers, Erlangen) equipped with a 32‐channel head coil. Multishell diffusion data were performed with a 2D spin‐echo single‐shot multiband EPI sequence (60 slices, repetition time = 3500 ms, 2 mm isotropic voxels, acceleration factor = 4). To optimize the acquisition for each shell, we minimized the echo time accordingly (Hutter et al., 2018). We used three shells with increasing diffusion directions (*b*‐value/direction/echo‐time = 1000/20/104, 2000/30/113, 3000/64/125) and three volumes without diffusion weighting (*b* = 0 s/mm^2^) at the beginning of each shell with the corresponding echo time. Additionally, to enable susceptibility distortion correction (Andersson et al., 2003), three b‐0 images were acquired for each shell with reverse phase‐encoding direction (posterior–anterior). A submillimetre 3D MPRAGE scan (224 slabs, time of repetition/echo time/time of inversion = 2400/2.26/1000 ms, 0.8 mm isotropic voxels, flip angle = 8°) was also obtained. Overall, the total time of acquisition was about 14 min. MRI data can be downloaded on OpenNeuro (https://doi.org/10.18112/openneuro.ds003138.v1.0.1).

### 5.2 MRI‐preprocessing and analysis

#### 5.2.1 Diffusion data preprocessing

All diffusion‐weighted images were preprocessed using MRtrix (version 3.0 RC3; Tournier et al., 2019). Data for each participant were visually inspected, and no data had to be discarded. The first steps in our preprocessing pipeline were applied on each shell separately because of the different acquisition parameters. Data were denoised with “dwidenoise” (Veraart et al., 2016). Next, motion correction, eddy current, and susceptibility‐induced EPI distortions were corrected (Andersson et al., 2017) with the opposite phase encoding direction. For this step, we used “dwipreproc”, which evokes FSL's *eddy*, *topup* and *applytopup*. Motion parameters were extracted and fed into a mixed ANOVA with factors for group and time. The results indicated no significant main effect for group (*F*(1,50) = 1.91, *p* = .173), time (*F*(2,100) = 1.4, *p* = .251), or the interaction between group and time (*F*(2,100) = 2.0, *p* = .131). All three shells were merged into a 4D file and corrected for intra‐session motion again with “dwipreproc,” but only motion correction was applied in this case. The last step in the preprocessing routine was a bias field correction based on the N4 algorithm (Tustison et al., 2010). Each T1‐weighted image was coregistered with the first b0 volume of the diffusion data for each participant and then segmented into five tissues (Smith et al., 2012) to enable multi‐tissue analysis.

#### 5.2.2 Fixel‐based analysis

For the fixel‐based analysis, we first computed a tissue response function for each participant for GM, WM, and CSF. Then, we separately estimated the average response function for the whole sample for the three tissue types. As recommended in the MRtrix documentation, we upsampled the data to 1.25 mm and computed a brain mask with this resolution. Next, multishell multi‐tissue (MSMT) spherical deconvolution was applied to estimate the fiber FOD. To make the absolute amplitudes of all subjects comparable, we performed a joint bias field correction and a global intensity normalization. Finally, we brought all DWI data into the same template space for later statistical analysis. This was achieved by creating a template of all subjects using the individual FOD images and then registering and warping all FODs to this FOD template (Raffelt et al., 2011). Next, each FOD lobe was segmented to identify the number and orientations of the corresponding fixels in each voxel. Fixels were then spatially reoriented into the template space, and matching fixels between individual and template fixels were identified. A whole‐brain probabilistic tractogram with 20 million streamlines was generated (Tournier et al., 2010) on the population template. This tractogram was filtered to 2 million streamlines using the SIFT (spherical‐deconvolution informed filtering of tractograms) algorithm (Smith et al., 2013). As the last step, the FD, FC, and the FDC were calculated for each participant and time point, all laying in the same template space.

#### 5.2.3 NODDI analysis

Neurite orientation dispersion and density imaging (NODDI) was performed based on the preprocessed data from the fixel analysis. We used the NODDI toolbox (v.1.04; Zhang et al., 2012) running under Matlab The MathWorks Inc. (2019). The NODDI model was fitted with the orientation‐dispersed cylinder model and Watson distribution, resulting in maps for the two following parameter estimates: (a) the NDI and (b) the ODI. Finally, NODDI parameters were brought into the fixel‐template space with the same transformation matrix as the fixel‐analysis. This should prevent any bias related to the normalization procedure.

#### 5.2.4 Tensor‐based analysis

To complement the DWI analysis, we performed a standardized tensor‐based analysis. Preprocessing steps were analog to the NODDI analysis, except we only used one shell (*b* = 1000). In short, preprocessed data (see Section 5.2.1) were inputted to the MRtrix‐command “dwi2tensor” to estimate the tensor for each voxel. Then, the following parameters were computed: FA, MD, axial diffusivity (AD) and RD. These parameters were also brought into the fixel‐template space.

### 5.3 Statistical analyses

#### 5.3.1 Fixel

Statistical inference was performed using fixel‐based analysis (FBA) implemented in MRtrix (Raffelt et al., 2011). For inferences following a repeated measurement design, it is necessary to prepare a permutation file to ensure that permutation testing is performed correctly. Therefore, we generated this file with a function (palm_quickperms) implemented in PALM (Winkler et al., 2014, 2015). Non‐parametric permutation testing was performed separately for each group using connectivity‐based fixel enhancement for FD, FC, and FDC. We focused on analyzing longitudinal changes employing paired *t*‐tests and did not compare groups explicitly. FBA results reported here were generated using 10.000 permutations and FWE‐corrected at *p*_FWE_ < .01.

#### 5.3.2 Tensor

NODDI‐ and tensor‐based parameters were analyzed in SPM12 (Wellcome Trust Centre for Neuroimaging, London, UK; https://www.fil.ion.ucl.ac.uk/spm/software/spm12). After smoothing the data with a minimal Kernel (FWHM = 2 mm), we built a flexible factorial model with the factors group (intervention, control) and time (pre, post, follow‐up) analog to the fixel‐analysis. Again, we were only interested in the within‐group changes and, therefore, computed contrasts for each time difference separately for each group. Finally, we used the TFCE toolbox (Smith & Nichols, 2009) to estimate a non‐parameter‐based statistical analysis and corrected all results at *p*_FWE_ < .01 after generating 10.000 permutations.

#### 5.3.3 Limitations

We employed a randomized intervention study design to examine WM plasticity. Participants were randomly assigned to either the intervention or CG. During the study planning phase, we aimed to ensure that a specific threshold of “lifetime” experience in slacklining was not surpassed. However, we did not impose strict inclusion criteria regarding age and sex. The CG exhibited a higher average age compared to the intervention group. Although we are confident that the modest mean age difference of 2.5 years in this study may probably be less of an issue, investigating the effects of age and sex more closely in future studies would be highly intriguing.

We used a specific acquisition protocol to optimize data quality and acquisition time for each shell in this study. However, it should be noted that varying echo times can potentially result in an overestimation within the NODDI model. Consequently, comparisons of our NODDI values with those from other studies should be cautiously approached. Nonetheless, our findings from a within‐study design remain unaffected, as statistical analyses are based on relative differences rather than absolute values. Future studies might benefit more from maintaining a constant echo time rather than optimizing each shell individually.

## CONFLICT OF INTEREST STATEMENT

The authors declare no conflicts of interest related to this study. No financial or personal relationships could have influenced the research outcomes.

## Supporting information


**Figure S1.** Fixel‐based interaction. Interaction between group and time for fiber‐cross section. Results are thresholded at *p*_FWE_ < .01 and color‐coded for direction.
**Figure S2.** Fixel‐based effect size. Changes of the slackline group for all three Fixel‐metrics between pre and post of. Only big effects (Cohen's *d* > .8) are reported. FC = red, FDC = yellow, FD = blue.
**Figure S3.** Increases of fiber density between pre and post. Thresholds are set *p* > .01 (FWE). Color represents the fiber‐direction.

## Data Availability

The data that support the findings of this study are openly available in openneuro.org at https://openneuro.org/datasets/ds003138/versions/1.0.1, reference number ds003138.
