# Supplementary material for: Tidying up white matter: Neuroplastic transformations in sensorimotor tracts following slackline skill acquisition
Source: Hum Brain Mapp. 2024 Nov 11;45(16):e26791. doi: 10.1002/hbm.26791 (PMC11551625; doi:10.1002/hbm.26791)
Supplement: Supplementary file 1 — Figure S1. Fixel‐based interaction. Interaction between group and time for fiber‐cross section. Results are thresholded at p FWE < .01 and color‐coded for direction. Figure S2. Fixel‐based effect size. Changes of the slackline group for all three Fixel‐metrics between pre and post of. Only big effects (Cohen's d > .8) are reported. FC = red, FDC = yellow, FD = blue. Figure S3. Increases of fiber density between pre and post. Thresholds are set p > .01 (FWE). Color represents the fiber‐direction. [file HBM-45-e26791-s001.pdf]

6 | APPENDIX

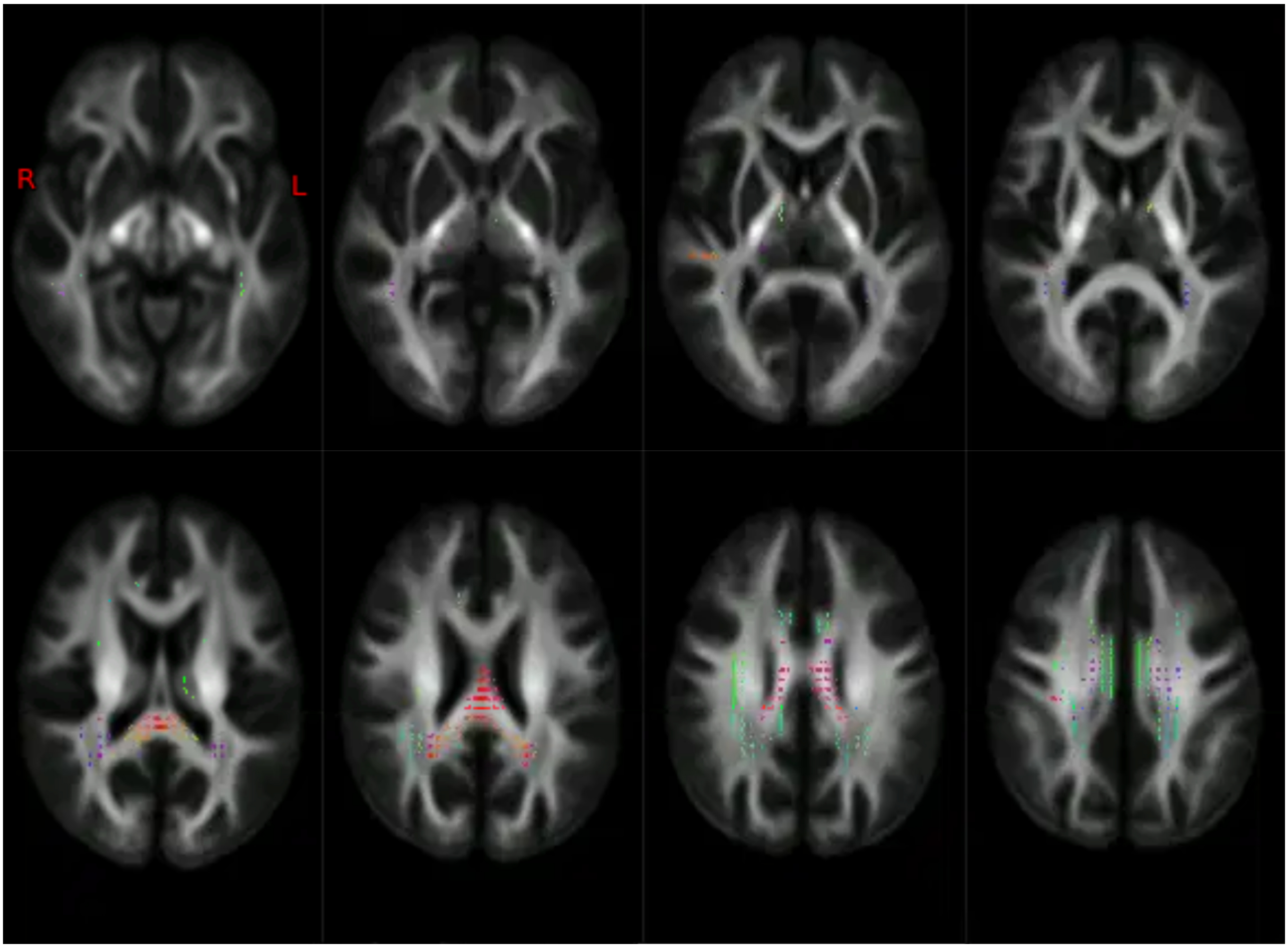

Supplementary Figure 1 Fixel-based interaction. Interaction between group and time for fibre-cross section. Results are thresholded at $p_{FWE} < 0.01$ and color-coded for direction.

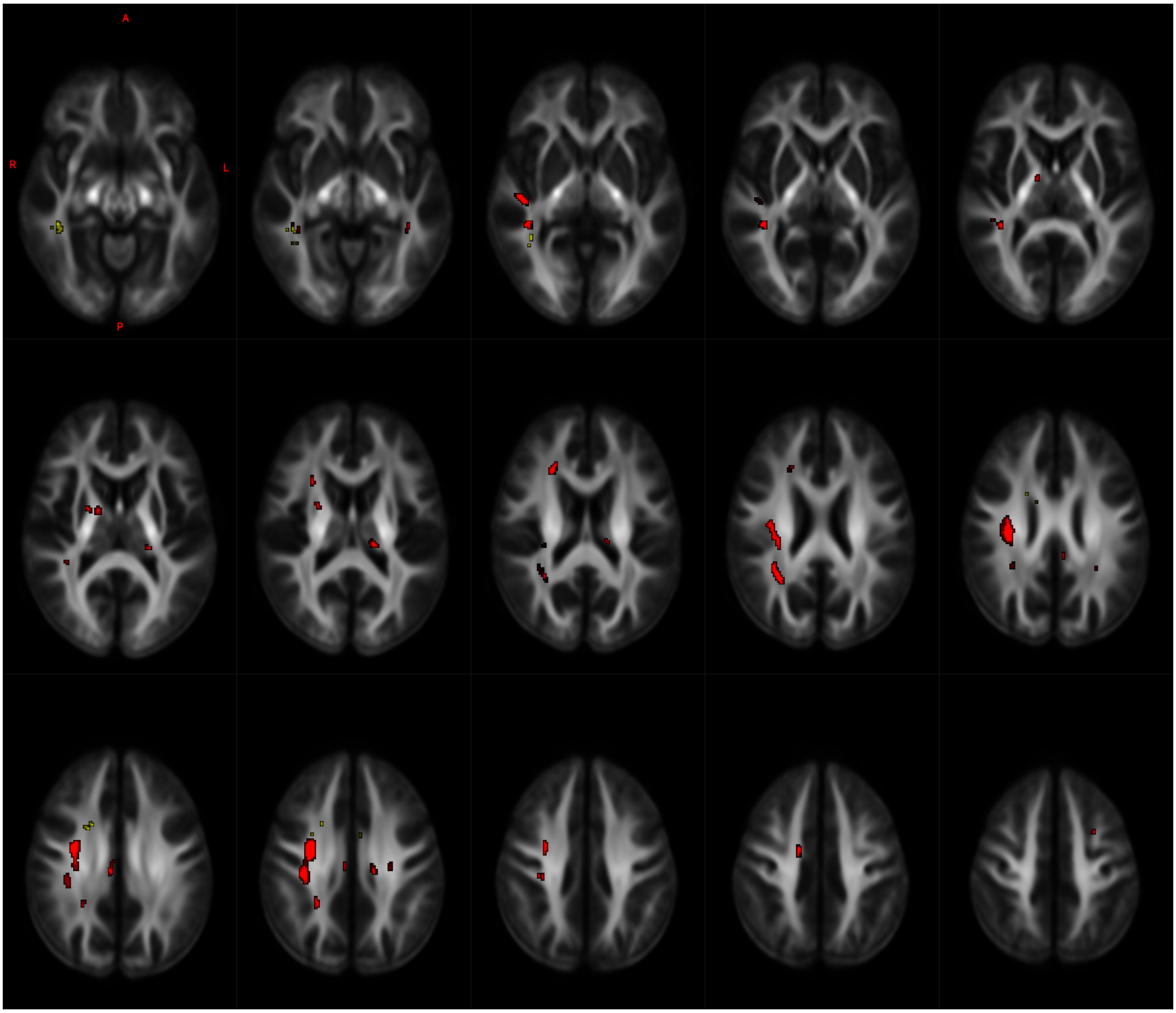

Supplementary Figure 2 Fixel-based effect size. Changes of the slackline group for all three Fixel-metrics between pre and post of. Only big effects (Cohen's> 0.8) are reported. FC = red, FDC = yellow, FD = blue

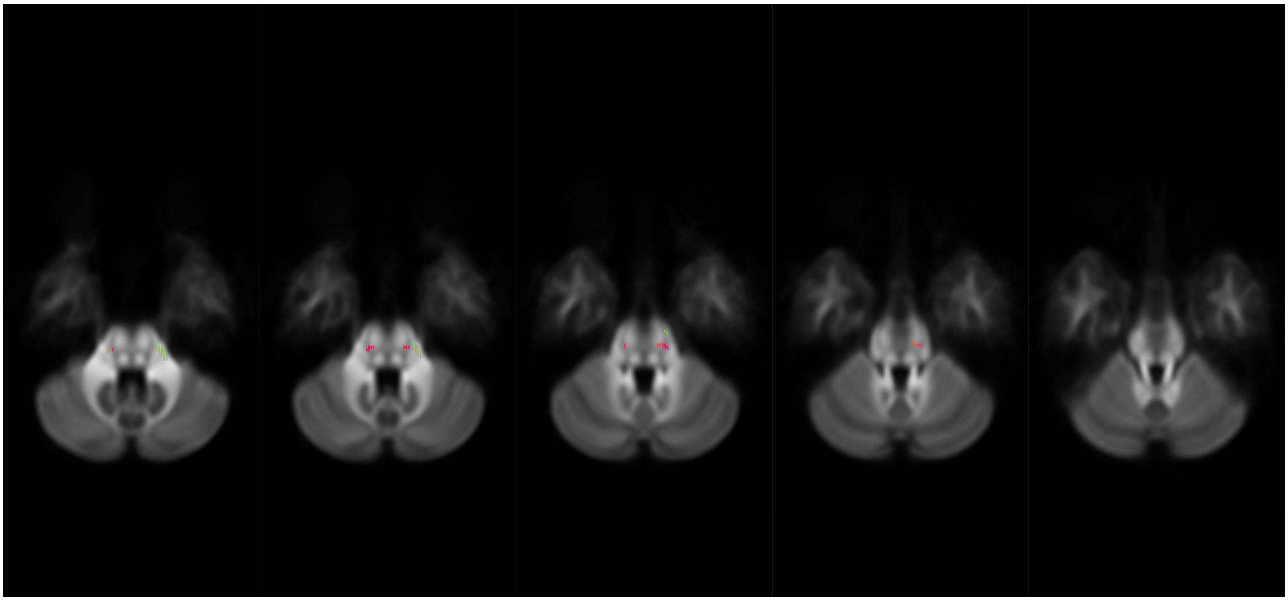

Supplementary Figure 3 Increases of Fibre density between pre and post. Thresholds are set p > 0.01 (FWE). Color represents fibre-direction.
